# 妊娠期免疫性血小板减少症患者在严重血小板减少情况下的出血特征

**DOI:** 10.3760/cma.j.cn121090-20250723-00345

**Published:** 2026-02

**Authors:** 燕梅 程, 仁霞 李, 志惠 冯, 诗琦 张, 丹丹 王, 会娟 敖, 伟 张, 泽平 周

**Affiliations:** 昆明医科大学第二附属医院血液内科，昆明 650101 Department of Hematology, the Second Affiliated Hospital of Kunming Medical University, Kunming 650101, China

**Keywords:** 妊娠, 免疫性血小板减少症, 严重血小板减少, 出血评分, 生理性高凝状态, Pregnancy, Immune thrombocytopenia, Severe thrombocytopenia, Bleeding score, Physiological hypercoagulable state

## Abstract

**目的:**

评估妊娠合并免疫性血小板减少症（Immune thrombocytopenia, ITP）患者在严重血小板减少（<20×10^9^/L）情况下，与同等血小板减少程度的非妊娠ITP患者相比，妊娠生理性高凝状态是否降低出血风险、减轻出血严重程度。

**方法:**

回顾性收集2013年1月1日至2024年4月30日期间，于昆明医科大学第二附属医院住院治疗的妊娠合并ITP患者（排除病理妊娠继发血小板减少患者）。并且根据年龄、ITP病程及PLT谷值3个标准，以1∶2的比例匹配非妊娠ITP患者，比较两组患者出血事件发生率及出血严重程度等。

**结果:**

本研究共纳入23例妊娠期间PLT<20×10^9^/L的ITP病例，匹配非妊娠ITP患者46例。妊娠ITP患者的活化部分凝血活酶时间（APTT）及凝血酶原时间（PT）较非妊娠ITP患者缩短（均*P*<0.05）。妊娠ITP组与非妊娠ITP组出血事件发生率（73.9％对89.1％，*P*＝0.161）及总体出血评分（*P*＝0.072）差异均无统计学意义。妊娠ITP组最高出血评分为5分，且仅有2例（8.7％），91.3％的病例出血评分≤3分。非妊娠ITP组最高出血评分为8分（危及生命的大出血事件），有3例（6.5％），1例失血性休克，2例脑出血（其中1例脑出血死亡）。19.6％的患者出血评分为5分，73.9％的患者出血评分≤3分。非妊娠ITP组出血评分≥5分的出血事件发生率是妊娠ITP组的3倍（26.1％对8.7％）。

**结论:**

患有ITP的妊娠期女性即使PLT<20×10^9^/L，与同等血小板减少程度的非妊娠ITP患者相比，其严重出血发生率降低，且妊娠ITP组APTT及PT时间更短，提示妊娠期的相对高凝状态可能是这部分患者出血严重程度减轻的潜在原因。

## Abstract (en)

**Objective:**

To evaluate whether the physiological hypercoagulable state of pregnancy affects the risk and severity of bleeding in pregnant patients with immune thrombocytopenia（ITP）and severe thrombocytopenia（PLT<20×10^9^/L）, compared to non-pregnant ITP patients with a comparable degree of thrombocytopenia.

**Methods:**

We retrospectively reviewed the data of pregnant patients with ITP who were hospitalized at the Second Affiliated Hospital of Kunming Medical University between January 1, 2013, and April 30, 2024, excluding patients with secondary thrombocytopenia due to pathological pregnancy. Non-pregnant ITP patients were matched as a control group at a 1∶2 ratio based on three criteria（age, ITP disease duration, and nadir PLT）, after which bleeding events and severity were compared between the two groups.

**Results:**

A total of 23 pregnant ITP patients with a PLT<20×10^9^/L during pregnancy were included and matched to 46 non-pregnant ITP patients. Pregnant ITP patients had significantly shorter activated partial thromboplastin time（APTT）and prothrombin time（PT）than non-pregnant ITP patients（both *P*<0.05）. No statistically significant differences were found between the pregnant and non-pregnant ITP groups in the incidence of bleeding events（73.9％ *vs* 89.1％, *P*＝0.161）or in their overall bleeding score（*P*＝0.072）. In the pregnant ITP group, the maximum bleeding score was 5, occurring in only 2 cases, 8.7％, while 91.3％ of cases had a bleeding score of ≤3. In the non-pregnant ITP group, 3 patients（6.5％）experienced life-threatening major bleeding（maximum score: 8）, comprising 1 case of hemorrhagic shock and 2 cases of cerebral hemorrhage, one of which was fatal. Additionally, 19.6% of patients in this group had a bleeding score of 5, and 73.9％ had a score of ≤3. The incidence of bleeding events with a score of ≥5 was three times higher in the non-pregnant group than in the pregnant group（26.1％ *vs* 8.7％）.

**Conclusion:**

Pregnant patients with ITP, even those with a PLT count <20×10⁹/L, have a lower incidence of severe bleeding compared to non-pregnant ITP patients with a comparable degree of thrombocytopenia; furthermore, they exhibit shorter APTT and PT, suggesting that the relative hypercoagulable state during pregnancy may be the potential reason for the reduced bleeding severity in these patients.

原发性免疫性血小板减少症（Primary immune thrombocytopenia, ITP）是最常见的获得性自身免疫性出血性疾病，其特征是由致病性抗血小板自身抗体、T细胞介导的血小板破坏和巨核细胞功能受损引起的孤立性血小板减少（外周血PLT<100×10^9^/L）[1]–[2]。妊娠期间ITP的发生率为1/1 000～1/10 000，可能在妊娠之前诊断，也可能在妊娠期间被新诊断出来。妊娠合并ITP是导致母亲在妊娠前中期PLT<50×10^9^/L的最常见原因[3]–[4]。既往有研究表明，由于孕期血浆容量增加导致的血液稀释作用以及血小板清除率的增加，许多正常孕妇的PLT低于非妊娠期，且在妊娠中期开始呈现逐渐下降趋势[5]。而在妊娠合并ITP患者中，血小板减少通常也随着妊娠的进程而进行性加重，在孕晚期达低谷，严重时可发生器官自发性出血[6]。然而，妊娠是一个独特的生理过程，在母体性激素水平增加和胎儿生长发育的影响下，母体的大多数凝血因子浓度增加，一些生理性抗凝蛋白减少，纤维蛋白溶解活性降低，出凝血平衡的天平向凝血增强的方向倾斜，形成一种生理性的高凝状态[7]。这种生理性的高凝状态可有效降低分娩时发生出血的风险，防止致命性出血事件的发生[7]。基于PLT≥20×10^9^/L的ITP患者自发出血的风险相对较低，在妊娠这种生理性高凝的状态下，PLT≥20×10^9^/L的妊娠ITP患者发生严重出血的风险极低[8]。但当PLT<20×10^9^/L时，出血风险会增加[9]–[14]，但妊娠高凝状态对血小板极低情况下患者出血倾向的影响尚不清楚，专家共识对这部分重点人群缺乏循证医学数据。本研究通过回顾性匹配研究对昆明医科大学第二附属医院妊娠ITP及非妊娠ITP患者的出血风险进行分析，旨在评估妊娠生理性高凝状态是否影响ITP患者血小板极低情况下的出血风险。

## 病例与方法

1. 研究对象：通过回溯昆明医科大学第二附属医院电子病历系统，纳入2013年1月1日至2024年4月30日期间于本院产科或血液内科住院的全部妊娠合并ITP患者（排除病理妊娠继发血小板减少患者）。根据年龄、ITP病程及PLT谷值3个指标，以1∶2的比例匹配非妊娠ITP育龄期女性。所有纳入研究的患者均符合《成人原发免疫性血小板减少症诊断与治疗中国指南（2020年版）》[15]中ITP的诊断标准（除外了病理妊娠继发血小板减少）。本研究为回顾性研究，已通过本院伦理委员会批准（伦理批准号：审-PJ-科-2024-285），并免除知情同意。

2. 数据收集：收集患者的一般临床资料，包括年龄、确诊ITP时的血小板计数、既往治疗、合并症、既往出血史、PLT谷值、出血评分、活化部分凝血活酶时间（Activated partial thromboplastin time, APTT）、凝血酶原时间（Prothrombin time, PT）、凝血酶时间（Thrombin time, TT）、纤维蛋白原（Fibrinogen, FIB）、国际标准化比率（International normalized ratio, INR）等。

3. 定义：①ITP出血评分根据中华医学会血液学分会血栓与止血学组发布的2016版ITP出血评分[16]。②既往出血史即为纳入本次研究前ITP病程中发生的最严重的出血事件。将出血严重程度分为轻度（无或仅有皮下出血）、中度（可见黏膜出血）、重度（深部器官或内部黏膜出血）、危重度（危及生命的出血）。因本次研究的患者均为育龄期女性，因此参照Rodeghiero等[17]发表的ITP出血评估标准，将月经增多不伴贫血评估为中度出血，月经增多伴贫血评估为重度出血。③严重出血即为出血评分≥5分的出血事件。

4. 统计学处理：采用SPSS 25.0软件进行统计学分析。计量资料如符合正态分布，则以均数±标准差（*x*±*s*）表示，两组比较使用独立样本*t*检验；若不符合正态分布，则以中位数及四分位数［*M*（*IQR*）］表示，两组比较采用Mann-Whitney *U*检验。等级资料的比较采用非参数检验。无序计数资料比较采用Fisher精确概率法。双侧*P*<0.05为差异有统计学意义。

## 结果

1. 患者特征：本研究共纳入23例妊娠ITP病例，并匹配46例非妊娠ITP病例。所有患者在出血事件发生前3个月内均未使用过抗血小板药物及抗凝药物。妊娠ITP患者与非妊娠ITP患者的人口学及ITP特征的比较详见表1，基线差异均无统计学意义。

**表1:** 妊娠ITP组与非妊娠ITP组的人口学及ITP特征比较

特征	妊娠ITP组（23例）	非妊娠ITP组（46例）	*P*值
入组年龄（岁，*x*±*s*）	28.61±3.24	27.52±6.25	0.345
ITP确诊时年龄（岁，*x*±*s*）	24.48±5.43	23.46±7.34	0.557
ITP病程［年，*M*（*IQR*）］^a^	4.33（0，6.58）	2.87（0.16，5.96）	0.898
ITP分期［例（％）］			1.000
新诊断	6（26.1）	12（26.1）	
持续性	1（4.3）	2（4.3）	
慢性	16（69.6）	32（69.6）	
既往治疗情况			
未治疗［例（％）］	7（30.4）	10（21.7）	0.429
累计药物治疗种类［种，*M*（*IQR*）］	1.0（0，2.0）	1.0（1.0，2.0）	0.607
激素［使用例数/总例数（％）］	15/16（93.8）	36/36（100.0）	
IVIG［使用例数/总例数（％）］	4/16（25.0）	12/36（33.3）	
TPO［使用例数/总例数（％）］	3/16（18.8）	6/36（16.7）	
泊帕类药物［使用例数/总例数（％）］	4/16（25.0）	10/36（27.8）	
其他药物［使用例数/总例数（％）］	5/16（31.3）	7/36（19.4）	
脾切除［例（％）］	0（0）	1（2.2）	1.000
PLT谷值［×10^9^/L，*M*（*IQR*）］	6.0（3.0，12.0）	4.5（1.0，11.0）	0.241
PLT谷值分组［例（％）］			1.000
<10×10^9^/L	16（69.6）	32（69.6）	
（10～<20）×10^9^/L	7（30.4）	14（30.4）	
ITP确诊时PLT［×10^9^/L，*M*（*IQR*）］	9.0（2.0，15.0）	8.0（3.0，12.8）	0.692
既往出血史［例（％）］^b^			0.126
轻度	10（43.5）	10（21.7）	
中度	10（43.5）	26（56.5）	
重度	1（4.3）	8（17.4）	
危重度	2（8.7）	2（4.3）	
合并症［例（％）］			
妊娠期高血压	2（8.7）	–	–
边缘性前置胎盘	1（4.3）	–	–
子宫肌瘤与腺肌病	1（4.3）	4（8.7）	0.658
呼吸系统感染	1（4.3）	8（17.4）	0.255
肝功能不全	1（4.3）	1（2.2）	1.000

**注** ITP：免疫性血小板减少症；IVIG：静脉注射免疫球蛋白；TPO：血小板生成素；–：不适用。^a^部分患者既往未曾诊断过ITP，多因出现出血症状首次来诊，完善检查后诊断的ITP，因此纳入时即为新诊断ITP，病程记录时间为0 d；^b^纳入本研究前ITP病程中发生的最严重的出血事件，轻度为无或仅有皮下出血，中度可见黏膜出血，重度为深部器官或内部黏膜出血，危重度为危及生命的出血

2. 凝血指标：妊娠ITP患者APTT［（31.38±4.64）s对（34.18±5.26）s，*P*＝0.034］及PT［12.10（11.40，13.10）s对13.05（12.38，13.78）s，*P*＝0.002］较非妊娠ITP患者明显缩短。而两组的TT［15.50（14.80，16.30）s对16.15（15.15，17.03）s，*P*＝0.201］、FIB［3.01（2.32，3.66）g/L对2.74（2.23，3.05）g/L，*P*＝0.101］以及INR［0.98（0.91，1.02）对1.01（0.95，1.09），*P*＝0.083］差异均无统计学意义（表2）。

**表2:** 妊娠ITP组与非妊娠ITP组的凝血功能及出血程度比较

变量	妊娠ITP组 （23例）	非妊娠ITP组 （46例）	*P*值
凝血功能			
PT［s，*M*（*IQR*）］	12.10（11.40，13.10）	13.05（12.38，13.78）	0.002
APTT（s，*x*±*s*）	31.38 ± 4.64	34.18 ± 5.26	0.034
TT［s，*M*（*IQR*）］	15.50（14.80，16.30）	16.15（15.15，17.03）	0.201
FIB［g/L，*M*（*IQR*）］	3.01（2.32，3.66）	2.74（2.23，3.05）	0.101
INR［*M*（*IQR*）］	0.98（0.91，1.02）	1.01（0.95，1.09）	0.083
出血事件［例（％）］^a^	17（73.9）	41（89.1）	0.161
出血评分［例（％）］^b^			0.481
0分	6（26.1）	5（10.9）	
1分	5（21.7）	11（23.9）	
2分	6（26.1）	10（21.7）	
3分	4（17.4）	8（17.4）	
5分	2（8.7）	9（19.6）	
8分	0（0）	3（6.5）	

**注** ITP：免疫性血小板减少症；PT：凝血酶原时间；APTT：活化部分凝血活酶时间；TT：凝血酶时间；FIB：纤维蛋白原；INR：国际标准化比率。^a^除外终止妊娠时及产后的出血事件；^b^根据中华医学会血液学分会血栓与止血学组发布的2016版ITP出血评分标准

3. 出血事件及出血评分：妊娠组中有17例（73.9％）发生出血事件（除外终止妊娠时及产后的出血事件），而非妊娠组中则有41例（89.1％）发生出血事件（*P*＝0.161）。妊娠ITP组及非妊娠ITP组出血评分见表2。其中妊娠ITP组最高出血评分为5分，且仅有2例（8.7％），高达91.3％（21/23）的病例出血评分≤3分，多为无出血或轻中度出血。而非妊娠ITP组最高出血评分为8分（危及生命的大出血事件），有3例（6.5％），其中1例失血性休克，2例脑出血（其中1例脑出血死亡）；出血评分为5分的非妊娠ITP病例有9例（19.6％），73.9％（34/46）的病例出血评分≤3分。非妊娠ITP组出血评分≥5分的出血事件发生率是妊娠组的3倍（26.1％对8.7％）。

## 讨论

妊娠对ITP患者是一个挑战，当患者在妊娠前或妊娠期间被诊断出患有ITP时，出血是最受关注的问题[6],[18]。迄今为止大多数研究提示妊娠期ITP患者出血风险低于非妊娠ITP患者。Webert等[19]收集了116例妊娠ITP患者的出血事件信息，65.5％的妊娠患者没有发生出血事件，12.9％的妊娠患者有轻微出血，18.1％的妊娠患者出血症状为中度，仅有4例（3.4％）妊娠患者的出血症状严重，其中2例血尿，1例血肿形成，1例胃肠道出血。但没有妊娠患者因出血症状而需要住院治疗。2006年Veneri等[20]对37例ITP患者的43次妊娠进行回顾性分析，其中39次妊娠完成分娩，在妊娠期间患者未发生出血并发症，在分娩期间仅发现3次（3/39，7.7％）出血并发症，出血并发症的发生率不足8％。2022年Gonzalez-Porras等[18]对ITP引起的妊娠相关出血并发症进行了一项Meta分析，其中共涉及了482例妊娠患者，其中113例发生出血事件，但大多数出血事件都是轻微的。仅有两项研究记录了严重出血事件，在其汇总的109例妊娠患者中，未报告任何此类事件发生[21]–[22]。提示ITP孕期大多数出血情况较轻，多为瘀点、瘀斑或中度失血。2023年Guillet等[23]分析了131例妊娠合并ITP患者及与之相匹配的131例非妊娠ITP患者的临床资料，比较其出血事件的发生率，结果显示妊娠与非妊娠两组患者之间严重出血事件的发生率差异无统计学意义，提示ITP患者在妊娠期间发生严重出血的风险仍较低。

但上述研究对象均来自西方人群。且一般认为包括中国患者在内的亚洲人群属于相对“易出血”体质。但目前尚无中国妊娠ITP患者出血程度的数据来回答。在本研究中，妊娠与非妊娠两组间的出血事件发生率及出血严重程度差异无统计学意义。但即使是在最需要关注的PLT谷值<10×10^9^/L的病例中，妊娠组ITP患者也未发生危及生命的出血事件，出血评分最高为5分，大部分出血事件为轻中度出血。而在同等PLT水平下，非妊娠组ITP患者发生危及生命的出血事件有3例，且其中1例死亡。非妊娠ITP组出血评分≥5分的出血事件发生率是妊娠组的3倍。由此可以看出，即使PLT<20×10^9^/L，妊娠组发生严重出血事件（即出血评分≥5分）的风险仍低于非妊娠组。

妊娠期孕产妇的高凝状态本质是一种生理性的适应状态，旨在降低分娩时的出血风险，提高母体生存率。而当ITP患者妊娠时，妊娠生理性高凝状态的变化可能会抵消ITP患者血小板减少导致的出血倾向，维持出凝血平衡，减少出血事件的发生。且在本研究中，我们通过匹配排除了年龄、ITP病程、PLT等危险因素对妊娠组与非妊娠组严重出血事件发生风险的影响。由此可推测在本研究中妊娠组发生严重出血事件的风险较非妊娠组低，与妊娠生理性高凝状态有关。我们进一步将妊娠ITP组与非妊娠ITP组的凝血指标比较，虽然两组的APTT及PT值均在正常值范围内，但妊娠组的APTT与PT值较非妊娠组缩短，提示妊娠ITP组未发生严重出血事件可能与妊娠ITP患者以APTT及PT缩短为表现的生理性高凝状态有关。妊娠期高凝状态为复杂的生理过程，仅使用APTT及PT评估孕妇高凝状态可能缺乏一定的准确性，但APTT及PT的缩短（即使在正常范围内）也能反应出内外源途径凝血因子的活性增强，提示凝血系统向促凝方向偏移。APTT与PT检测操作简便及成本低，目前在实际临床工作中仍被广泛使用。

目前关于妊娠ITP的治疗指征主要基于专家意见和少量回顾性研究的结果，且多参考非妊娠成人ITP的诊疗。2019年更新版ITP国际共识[24]认为，如果妊娠期PLT稳定在（20～30）×10^9^ /L、无出血症状的患者可以观察；为防止严重出血事件的发生，PLT<20×10^9^ /L时需要接受治疗。而在我们的研究中发现，即使PLT<20×10^9^ /L，与同等血小板水平的非妊娠ITP患者相比，孕妇的严重出血风险较非孕妇的严重出血风险低。那我们是否可以进一步降低妊娠ITP患者开始接受治疗的血小板阈值至10×10^9^ /L，或无论血小板水平如何，患者如无出血症状则予严密监测出血倾向。我们的研究为下调妊娠ITP患者治疗起始标准提供了初步依据。值得进一步通过前瞻性研究进行证实，以避免妊娠期ITP患者被过度治疗，同时降低药物对妊娠期女性及胎儿的风险。

然而本研究存在局限性，结果应谨慎解读。首先本研究为单中心回顾性研究，样本量较少，虽然妊娠组与非妊娠组间按年龄、病程及PLT谷值3个标准进行了匹配，但数据收集可能存在偏倚。其次，我们仅收集了妊娠ITP患者PLT<20×10^9^/L期间的出血事件，未收集其产后出血事件的数据。

综上所述，我们的研究结果表明，患有ITP的妊娠期女性即使PLT<20×10^9^/L，与同等血小板减少程度的非妊娠ITP患者相比，其严重出血发生率降低，同时凝血功能显示妊娠ITP组APTT及PT时间更短，提示妊娠期的相对高凝状态可能是这部分患者出血严重程度减轻的潜在原因。该研究首次提供了中国妊娠ITP患者的出血程度数据，为评估这部分人群的风险及启动治疗时机提供了依据，后续需要样本量更大的多中心、前瞻性研究进行进一步验证。
